# Supplementary material for: A nutrient-sensitive enterokine coordinates developmental plasticity through inter-organ signaling
Source: PLoS Biol. 2026 Aug 11;24(8):e3003911. doi: 10.1371/journal.pbio.3003911 (PMC13460628; doi:10.1371/journal.pbio.3003911)
Supplement: Supplementary file 1 [file pbio.3003911.s013.docx]

**Editor statement**

The reviews are attached below. As you will see, the reviewers find the conclusions interesting and worth pursuing, but they also raise several issues that would need to be addressed, some of them experimentally.

Reviewer 1 has several suggestions to improve the figures and also thinks the controls for genetic background should be discussed, along with the limitations in the discussion.

**We have addressed Reviewer #1’s suggestions by improving both the presentation and discussion of our findings. Specifically, we added additional figure panels showing full images of the corpora cardiaca and midgut to provide clearer spatial context for Lst expression patterns. We also refer to the conceptual model figure in the main text. We also clarified that the RNA-seq dataset and complete statistical analyses had been previously published and deposited in GEO, and we now explicitly reference these resources in the manuscript.**

**In response to concerns regarding genetic background controls, we substantially expanded the Methods section to detail the rationale and use of matched control lines for each RNAi background (VDRC KK, VDRC GD, and TRiP collections), ensuring appropriate interpretation of the RNAi experiments.**

**Finally, following the reviewer’s recommendation, we expanded the Discussion to better acknowledge the limitations of the Gal4-based approaches and the possibility that additional low-level or dispersed Lst-expressing cells may contribute to the phenotype. We now more carefully discuss the interpretation of the identified anterior midgut Lst^+^ EECs as a candidate signalling center, while explicitly recognizing that additional local sensing mechanisms or other contributing cell populations cannot currently be excluded.**

Reviewer 2 asks for a better description of the developmental stage of the animals and mentions that an alternative explanation for the EECs regulation should be considered, and test whether constitutive activation of TOR signalling in the fat body is sufficient to block lst induction upon AA restriction.

**In response to Reviewer #2, we have clarified throughout the revised manuscript that larvae were collected using a stage- and size-matched strategy (L3 larvae, ~3.5 mm length) to ensure biologically meaningful comparisons despite differences in developmental timing under the various nutritional and genetic conditions. These details have now been explicitly added to the Methods section.**

**We also addressed the reviewer’s suggestion that EECs may directly sense amino acid availability. Specifically, we tested the role of the amino acid transporter slif in EECs and found that neither *slif* expression nor EEC-specific *slif* knockdown significantly affected gut-derived *Lst* induction under amino acid restriction. While these results do not support a primary role for slif, we now discuss the possibility that other amino acid transporters could contribute to cell-autonomous nutrient sensing in EECs.**

**Finally, we tested whether constitutive activation of TOR signalling in the fat body could suppress *Lst* induction under amino acid restriction by overexpressing *Rheb*. This manipulation caused strong developmental defects and lethality and did not suppress *Lst* expression, making the results difficult to interpret in the context of physiological nutrient sensing. As these findings are peripheral to the central conclusions of the study and do not provide a clear mechanistic interpretation, we chose not to include them in the revised manuscript, although we provide them to the reviewer for evaluation.**

Reviewer 3 mentions that Lst positive EEC activity or the haemolymph hormone should be assessed to strengthen the results, and confirm that these EECs don’t respond to dietary sugars, adding missing controls, among other points.

**In response to reviewer #3 comments, we performed several new experiments and added multiple control conditions that substantially strengthen the revised manuscript.**

**To directly address the concern regarding Lst protein production and EEC activity, we performed anti-Lst immunostaining and detected Lst protein in the identified EEC population. Because steady-state intracellular peptide levels may not accurately reflect secretion dynamics, we additionally blocked vesicle-mediated secretion in EECs using targeted expression of *shibire^ts^*. Under these conditions, Lst protein strongly accumulated in EECs, with markedly higher accumulation observed in undernourished germ-free larvae compared to normally fed controls. These new experiments support the conclusion that the transcriptional induction of *Lst* is accompanied by increased Lst protein production and secretion.**

**We also addressed the reviewer’s concern regarding nutrient specificity. Using holidic diets with controlled nutrient composition, we found that gut-derived *Lst* expression is highly sensitive to amino acid restriction, whereas moderate reduction of dietary sucrose had no significant effect. Only complete sugar deprivation mildly induced *Lst* expression, indicating that Lst-positive EECs are primarily sensitive to amino acid availability.**

**In addition, we incorporated several missing controls and validation experiments throughout the revised manuscript, including:
(i) protein-level validation of *Lst* RNAi lines and antibody specificity,
(ii) additional developmental timing controls under high amino acid diet conditions,
(iii) new epistasis experiments linking systemic insulin signalling to the EEC InR/FoxO/Lst axis,
and (iv) additional analyses of Ilp2 accumulation and systemic insulin signalling upon EEC-specific *Lst* manipulation.**

**Together, these additions significantly reinforce the mechanistic framework and central conclusions of the study.**

Finally, Reviewer 4 asks for additional discussion of the specificity of the response to LpWJL.

**In response to Reviewer #4, we expanded the Discussion and added new experimental data demonstrating that the regulation of gut-derived Lst is not specific to *Lp^WJL^*. In addition to *Lp^WJL^*, we found that both another *Lactiplantibacillus plantarum* strain (*Lp^NC8^*) and *Acetobacter pomorum^WJL^*, a distinct core member of the *Drosophila* microbiota, similarly suppress *Lst* expression under amino acid malnutrition conditions. These findings support a model in which gut-derived Lst responds broadly to microbiota-derived amino acid rather than to other strain-specific bacterial signals.**

**We also expanded the Discussion to better place this mechanism in an ecological context, emphasizing that *Drosophila* larvae naturally encounter fluctuating nutrient environments in decaying fruits, where microbiota-derived amino acids can critically support larval growth and developmental adaptation.**

**Reply to Reviewer #1**

Reviewer #1:

Bai et al discover a role of anterior midgut-localized Limostatin-producing cells in the regulation of *Drosophila* larval development by using a malnutrition paradigm that more closely resembles low nutrient conditions in the wild. They find that Limostatin represses developmental progression, conditional on the nutrient composition of the growth medium, with low nutrients causing upregulation of Lst and slowing of development. The authors find that the bacterium *Lp^WJL^* is sufficient to inhibit Lst production, consistent with its role in providing additional nutrients to the malnutrition medium. While Limostatin is known as a decretin in adults, the role in larval development has only been presumed, and the localization of Lst-producing cells has been thought to be in the corpora cardiaca. The authors use Gal4 drivers and antibodies to localize and characterize the functional importance of the new cell population that they discovered in the midgut. The paper is very clearly written and the data well-presented. I think the evidence is strong enough for publication.

**We thank Reviewer #1 for the positive and encouraging assessment of our manuscript.**

**We have addressed all the following minor points, which we believe have improved the clarity and refinement of our manuscript.**

I have some minor comments that I think the authors should address through writing changes in the manuscript.

1. Fig. 1. Since the unbiased RNAseq approach was used to identify Lst as an interesting candidate, a full presentation of the data would provide readers with a more unbiased perspective on the data. A volcano plot or other depiction of the complete dataset would suffice. DESeq2 could be used for the statistical test. If the data are messy or there are many other genes that are differentially upregulated in GF vs *Lp* colonized flies, this additional analysis could be put in the supplement and the rationale for pursuing Lst could be elaborated.

**The RNA-seq dataset referenced in this study was previously published in our recent work (Bai *et al*., Development, 2026; doi:10.1242/dev.204909), in which we showed that association of germ-free animals with Lactiplantibacillus plantarum strain WJL (*Lp^WJL^*) selectively promotes midgut growth relative to other organs in undernourished Drosophila larvae. In that published study, we used the dataset to investigate *Lp*-mediated modulation of local Ecdysone signalling and its downstream target genes, and demonstrated that this pathway is required locally for both intestinal and systemic growth. During further analysis of the same dataset, Lst (*CG8317*) emerged as a high-priority candidate gene for follow-up studies, as it was significantly downregulated upon *Lp^WJL^* colonization, as shown in the volcano plot of our previous work (Fig. 2b in Bai *et al*., 2026). Given the previously reported role of Lst as a decretin hormone in adult Drosophila (Alfa et al. Cell Metabolism 2015), this regulation made it a particularly compelling candidate for functional characterization in the context of nutrient-dependent developmental growth adaptation. Accordingly, the complete RNA-seq dataset and statistical analyses have already been published in Bai *et al.* (Development, 2026) and deposited in the NCBI Gene Expression Omnibus (GEO) database under accession number GSE310159. We have now clarified our text (line 93-95) and added this information to the legend of Figure 1.**

2. Fig. 2. (A+B) please show zoomed out images of the entire dissected CC and gut for spatial reference.

**Following this recommendation, we have now included representative images of the entire dissected corpora cardiaca and midgut in Fig. S2A–B, which serve as a spatial reference for the localized expression patterns described in the main figures.**

3. Please discuss the controls for genetic background in the main text of the paper. For example, when a Gal4 line and a UAS line are crossed, were controls used for each background? In Fig. 3 for instance, there is a Control 1 and a Control 2 in some panels. Please explain these further and provide the rationale in the main text or methods. I think it is particularly important for trainees who read *Drosophila* papers to appreciate how genetic background is accounted for experimentally -- either with backcrossing, with measurements of the parental phenotypes, or with inducible mutations. I think it would also be helpful to discuss the limitations in the discussion.

**We agree that rigorous control for genetic background is paramount in *Drosophila* research. Accordingly, in all experiments, we utilized controls that closely match the genetic background of the experimental RNAi lines.**

**For example, in Fig. 4, the *Lst* RNAi line from the VDRC KK library (VDRC#106861) contains two insertion sites (at 30B and 40D). To account for this, we used two independent control lines (VDRC#60102 and VDRC#60103) provided by VDRC for the KK library. These control lines utilize the same host strain background and contain UAS-driven inverted repeats of a GFP sequence inserted into the same respective landing sites (30B or 40D). This ensures that any observed phenotypes are not due to the site of insertion nor the expression of RNA hairpins nor the activity of the RNAi machinery itself. For *Lst* RNAi using TRiP lines from the BDSC, we primarily utilized the standard TRiP RNAi control line expressing *mCherry* dsRNA (BDSC#35785). To maintain consistency in experimental design with our KK line assays, we included an additional control background line (BDSC#36304) for this *Lst* TRiP RNAi line. Similarly, for VDRC GD library, we used the recommended background line (VDRC#60000) as the control.**

**In all instances, the same Gal4 driver line was crossed to both the experimental RNAi line and its respective controls to ensure the genetic contribution of the driver was identical across groups. We appreciate the review for pinpointing this; as recommended, we have now provided more details in the Method section.**

4. I'm not fully convinced that the small subset of cells in the anterior midgut that show high Lst expression are a signaling center composed of the only causal cells for the fly developmental phenotype. The authors acknowledge that the Gal4 drivers could have off-target effects, and I appreciate that. Could these cells just be mirroring the effect from the causal cells, which could be located in another region that the Gal4 driver targets? Could there be many dispersed cells expressing Lst at lower levels to produce the organismal growth effect? I am not asking for additional experiments but think that the Gal4 caveats and their implications should be discussed a bit more.

**We thank the reviewer for raising these insightful concerns and agree that the sparse population of Lst-expressing cells warrants careful interpretation. During the early stages of this project, we were similarly surprised by the restricted pattern of *Lst* expression. We therefore validated our *Lst*-Gal4 reporter results using a newly generated polyclonal anti-Lst antibody, which consistently detected Lst protein in the same discrete population of anterior midgut cells, with no additional staining beyond the previously described corpora cardiaca Lst+ cells. It is important to note that the larval midgut contains relatively few enteroendocrine cells (EECs) compared to the adult midgut (~100–200 total EECs). These larval EECs are embryonically derived and do not substantially increase in number during larval life. Thus, the limited number of Lst+ cells observed is consistent with the overall organization of the larval enteroendocrine system. That said, we acknowledge that the currently available tools may not detect cells expressing very low levels of Lst. Therefore, we cannot formally exclude the possibility that additional dispersed cells expressing Lst at lower levels also contribute to the systemic growth phenotype. However, our RNAi analyses using multiple drivers (*EEC*-Gal4, *AKH*-Gal4, and *Lst*-Gal4) consistently indicate that Lst expression within EEC Lst+ cells is required for adaptation to malnutrition. While contributions from other cell populations cannot be entirely excluded, these experiments support the conclusion that the identified EEC population plays an essential functional role. We now discuss these caveats and their implications in the revised manuscript (lines 381-386).**

5. I am curious from an evolutionary biology perspective why the effective Lst is expressed specifically in the EECs in the anterior midgut rather than in another signaling center such as the CC? Is there a potential integration of the local nutrient status in the gut with systemic nutrient status? Why should it work this way versus in an alternative way? Is there a particular developmental reason based on the cell lineages? Some discussion of these questions would strengthen the manuscript.

**Our study emphasizes a systemic regulation of *Lst* expression via Ilp2 signalling, despite *Lst* being expressed in EECs. We therefore propose that the gut functions here as an endocrine organ integrating organismal nutritional status, rather than solely as a local sensor of luminal nutrient content (a hypothesis we partly ruled out with our experimental manipulations of canonical amino acid sensors (mTOR and Gcn2) and the amino acid transporter slif within the EECs). In this context, the small population of Lst^+^ cells may indeed function as a signalling center, analogous to the IPCs, which also comprise only a few pairs of neurosecretory cells yet exert profound systemic effects on growth and metabolism. We propose that these Lst^+^ EECs act as mediators in a feedforward loop that ensures developmental robustness. At the same time, we agree with the reviewer that such a centralized signalling role does not exclude the existence of additional local sensing activities that may not be captured in the present study. We now discuss this possibility in the revised manuscript (lines 419-426).**

I think it would be worth including the model figure in the main text if there is room.

**We agree that a model figure is essential for synthesizing our findings. We have moved the conceptual model to the main Figure 9.**

line 207: in gut --> in the gut

line 208: majors --> major

**We have corrected these typos.**

**– – – – – –**

**Reply to Reviewer #2**

Reviewer #2: Fumiaki Obata – note that this reviewer has signed the review.

In this study, the authors address the endocrine mechanisms of adaptation to malnutrition in *Drosophila* larvae. They identified a previously unrecognized role of the peptide hormone Limostatin (Lst) in regulating larval developmental plasticity in response to amino acid (AA) availability. While Lst has been characterized primarily as a hormone produced by the corpora cardiaca in adult flies, this work convincingly demonstrates that, in larvae, Lst is secreted from a small population of midgut enteroendocrine cells. Through the use of chemically defined diets and well-controlled tissue-specific genetic manipulations, the authors delineate an inter-organ endocrine circuit linking AA sensing to systemic insulin signaling and developmental progression. The experiments are carefully executed, and the data consistently support the proposed model. Overall, this is a clearly presented study that provides new insight into how nutritional status is integrated at the organismal level to regulate developmental pace.

I have only minor comments, which the authors may find helpful for improving the manuscript. Addressing these points would further strengthen the study, but additional experiments are not strictly required.

(Fumiaki Obata)

**We would like to sincerely thank the reviewer for this constructive and encouraging evaluation of our work. We appreciate the recognition of our efforts to delineate the inter-organ endocrine circuit linking gut-derived Limostatin to systemic growth control. We have carefully addressed each comment which we agree have significantly improved the clarity and impact of our manuscript. Our point-by-point responses are provided below.**

1. The developmental stage of animals are not well described. It might be better to clearly specify the developmental stage of larvae, especially given that dietary conditions or Lst manipulation are expected to alter developmental timing. Were samples collected at matched chronological time points or at matched developmental stages?

**We acknowledge that both dietary conditions and *Lst* manipulation significantly influence *Drosophila* larval growth and developmental timing. To ensure biological comparability across groups with different growth rates, we adopted a stage and size-matched sampling strategy for our *Drosophila* experiments. Specifically, the developmental progression of larvae under normal feeding is approximately 2–3 days faster than that of *Lp*-associated undernourished larvae, and 5–6 days faster than that of germ-free undernourished larvae. By collecting stage- (L3) and size-(3.5mm) matched larvae, we minimized the confounding effects of size and physiological age, ensuring that our comparisons reflect the underlying biological mechanisms rather than mere developmental delay. As suggested, we have updated the Methods section to clearly specify the developmental stages.**

2. While the proposed model emphasizes an indirect relay via the fat body for the regulation of EECs, it is also possible that EECs directly sense amino acids (independently of GCN2/TOR). For example, does *slif* RNAi in EECs induce lst expression? In the model, there might be a direct arrow from "Low AA" to EEC. In addition, it would be informative to test whether constitutive activation of TOR signaling in the fat body (e.g. by *Rheb* overexpression) is sufficient to block *lst* induction upon AA restriction.

**We appreciate the reviewer’s suggestion to explore potential direct amino acid sensing within the EECs. To investigate whether amino acid transporters contribute to direct amino acid sensing in EECs, we first examined *slif* expression in the midgut using cDNA samples from larvae fed a holidic diet. Our results indicate that *slif* expression levels are not significantly altered by amno acid malnutrition (see Figure R1A below). Furthermore, targeted knockdown of *slif* specifically in EECs did not significantly induce gut-derived *Lst* expression (Fig. R1B). While these data suggest that slif is not a primary mediator in this context, we acknowledge the reviewer's point that, given the presence of over 20 amino acid transporters encoded in the *Drosophila* genome, a cell-autonomous sensing mechanism involving alternative transporters cannot be entirely ruled out, we have added this possibility in our manuscript (line 421-423).**

**Regarding the role of the fat body, we tested whether constitutive activation of TOR signalling could prevent *Lst* induction during amino acid restriction by overexpressing *Rheb* in the fat body using the *Lpp*-Gal4 driver. Unexpectedly, *Rheb* overexpression did not suppress *Lst* expression. Instead, surviving larvae displayed a tendency toward increased *Lst* expression together with developmental delay and reduced systemic growth (Fig. R1C–D). In addition, this manipulation caused high larval lethality. This phenotype is consistent with previous studies showing that ectopic activation of TOR signalling in the fat body under starvation conditions can impair the normal metabolic adaptation to nutrient deprivation, notably through inhibition of autophagy and defective mobilization of internal nutrient stores (Scott et al., *Developmental Cell*, 2004). Such dysregulated mTOR activity has also been associated with increased starvation sensitivity and impaired systemic growth coordination (Patel et al., *Journal of Cell Science* 2006). We therefore interpret these results cautiously, as constitutive Rheb activation under nutrient restriction appears to generate a pathological physiological state rather than selectively mimicking nutrient sufficiency signalling. Under these conditions, secondary systemic effects may indirectly promote *Lst* expression despite elevated mTOR activity in the fat body. Because these results do not provide a straightforward interpretation and are peripheral to the central conclusions of the study, we have chosen not to include them in the revised manuscript, although we are happy to provide them to the reviewer.**

**Figure R1. Effects of slif-mediated amino acid transport and TORC1 signaling on gut-derived *Lst* expression and developmental timing**

**(A) Relative transcript levels of gut-derived *slif* analyzed by qRT-PCR in germ-free (GF) larvae reared under low amino acid holidic diet (LAAHD) (*n* = 6).**

**(B) Relative transcript levels of gut-derived *Lst* analyzed by qRT-PCR in undernourished GF larvae with EEC-specific *slif* knockdown (*EEC>UAS-slif* RNAi) compared to their respective GD controls (*n* = 5).**

**(C, D) Relative transcript levels of gut-derived *Lst* analyzed by qRT-PCR (C) and median time to pupariation measured (D) in undernourished GF larvae with fat body (FB)-specific *Rheb* overexpression (*Lpp>UAS-Rheb*) compared to their respective overexpression controls. *n* = 6 (C), *n* ≥ 11 (D).**

**In (A–C), data are presented as mean ± SEM. Statistical significance was determined using a two-tailed unpaired *t* test (A, B, D) or a two-tailed Welch’s *t* test (C). Exact *P* values are indicated on the respective panels.**

3. In the model, FoxO is directly regulating *lst* expression. Is there evidence supporting this, such as the presence of FoxO-binding motifs in the regulatory region upstream of the *lst* gene?

**Our functional experiments demonstrate that FoxO is both necessary and sufficient to induce gut-derived *Lst* expression under malnutrition conditions. To address the reviewer’s comment, we analyzed *Lst* genomic locus sequence for potential FoxO response elements using the consensus binding sequences previously described by Spellberg et al. PNAS, 2015. This analysis identified three putative FoxO-binding motifs within the 2 kb proximal promoter region of *Lst*, as well as an additional consensus motif located within an intronic region. We have added this information to the revised manuscript and included a schematic representation in new Fig. S7. While the presence of these motifs does not formally demonstrate direct binding, these sequence analyses are consistent with a model in which FoxO directly regulates *Lst* transcription in response to nutritional stress.**

4. Given that only ~10 EECs express *lst*, further characterization of this population would be of interest. For example, analysis of available single-cell RNA-seq datasets could reveal whether these cells express additional distinctive markers or signaling components.

**We agree that further molecular characterization of this rare EEC subpopulation would be highly informative and represents an important direction for future work. We explored the possibility of leveraging available single-cell RNA-seq resources; however, currently available datasets do not provide sufficient resolution or coverage of *Drosophila* larval enteroendocrine cells to confidently identify and characterize the small population of Lst-expressing cells. We therefore believe that dedicated high-resolution single-cell profiling of larval EECs will be required to define the molecular identity of these cells and to identify additional markers and signalling components associated with the Lst-positive population.**

5. Please check the notation of Limostatin throughout the manuscript, as there are inconsistencies in several figures.

**We apologize for the inconsistencies in the notation of Limostatin. We have thoroughly audited the text and figures to ensure that the gene (*Lst*, italicized) and protein (Lst, non-italicized) symbols are applied consistently throughout the manuscript in accordance with standard nomenclature.**

**– – – – – –**

**Reply to Reviewer #3**

Reviewer #3:

This manuscript by Bai et al describes the presence of a midgut enteroendocrine source of the hormone Limostatin (Lst), which regulates organismal developmental control in response to dietary nutrients (AA in particular) and the microbiota in *Drosophila* larva.

The expression of Lst in the larval midgut is regulated by nutrients through Insulin signaling and it, in turn, controls Insulin signal production by the brain. The authors propose that low nutrient condition in the absence of microbiota leads to low insulin signaling in enteroendocrine cells (EECs) which, through FOXO, induces expression of Lst in EECs. Lst in turn further inhibits brain Ins.

This is, to my knowledge, the first report on a functional role of the larval midgut and EECs in the regulation of whole-body physiology in Drosophila. As such, this work presents exciting findings of great conceptual novelty and biological significance.

However, there is critical work that needs to be done, including important controls, to fully support the main claims of the study and to also strengthen its significant and impact in the field. I believe the suggested work below is also important to bring the study into publishable form.

**We thank Reviewer #3 for their insightful comments and for recognizing the conceptual novelty and biological significance of our work.**

**We have addressed the reviewer’s concerns through new experimental data, additional control conditions, and an expanded Discussion that incorporates these new findings into our model. We believe these revisions have significantly enhanced our findings, and we provide detailed, point-by-point responses to each specific comment below.**

1- Regulation of by dietary amino acids, microbiota and Ins signaling.

In most, if not all cases, throughout the manuscript, this is concluded from assessment of mRNA expression of Lst in whole midguts. This work needs to be complemented by the used of antibody and immunostaining to visualise the meaning of the mRNA levels observed. Also, levels of protein in the gut need to be followed by either assessment of Lst positive EEC activity (for example thorugh CaLexA of GCamp signaling) or assessment of haemolymph hormone, which is very difficult, so I suggest the former. Only then, the authors will be able to conclude what is happening with Lst in the various conditions studied.

**We agree that validating our transcriptional analyses at the protein level is important. While direct quantification of circulating Lst in the hemolymph would be ideal, this remains technically very challenging because the mature Lst peptide is predicted to be only 15 amino acids in length and is likely present at extremely low concentrations within the limited volume of larval haemolymph. To address this point, we performed complementary protein-level analyses using an anti-Lst antibody. We detected Lst protein in the relevant EEC population; however, differences in steady-state intracellular staining between conditions were less pronounced than the corresponding changes observed at the mRNA level (Fig. 3A). We interpret this cautiously, as Lst is a secreted peptide hormone and intracellular accumulation may not accurately reflect rates of synthesis and secretion, similarly to what has been described for dIlp2 in IPCs.**

**Regarding the reviewer’s suggestion to monitor EEC activity using CaLexA or calcium reporters, we reasoned that these approaches would primarily report global EEC activity rather than Lst-specific secretion dynamics, since individual EECs typically co-express several peptide hormones. We therefore adopted a more direct approach aimed at visualizing intracellular accumulation of Lst by acutely blocking vesicle-mediated secretion. Specifically, we expressed the temperature-sensitive dynamin allele (*shibire^ts^*) in EECs and shifted larvae to the restrictive temperature for 3 hours to inhibit secretion. Under these conditions, we observed a marked intracellular accumulation of Lst protein, which was substantially stronger in undernourished germ-free larvae than in normally fed controls (Fig. 3B).**

**Together, these experiments support the conclusion that the induction of *Lst* mRNA under malnutrition is accompanied by increased Lst protein production and secretion, consistent with our proposed model.**

2- EEC Limostatin has a developmental only role.

As part of their conclusions, the authors propose that Lst from EEC works in development, but the CC hormone works in adults. Have they looked at the expression or role of Lst in adult EECs at all?

**We thank the reviewer for this insightful question regarding the stage specificity of gut-derived Lst. To address this, we examined the adult midgut using the *Lst-Gal4>UAS-GFP* reporter line and did not detect any GFP-positive cells. Consistent with this observation, qPCR analysis showed negligible expression of *Lst* in the adult midgut (Fig.R2), a finding that aligns with publicly available adult single-cell RNA-seq datasets (e.g., Fly Cell Atlas). Furthermore, unlike the significant induction observed in larvae, malnutrition failed to significantly stimulate *Lst* expression in the adult gut (Fig.R2). These data support our model that the midgut is a larval-specific source of Lst. While gut-derived Lst plays a critical endocrine role in larval development, this endocrine regulation appears to be absent at the adult stage, where Lst production is primarily localized to the corpora cardiaca. We have now added a mention to the lack of adult Lst^+^ EECs (line 142).**

**Figure R2. Nutritional effect on gut-derived *Lst* expression in adult flies**

**(A) Relative transcript levels of gut-derived *Lst* analyzed by qRT-PCR in germ-free adult flies under either normal fed or undernourished conditions (*n* = 3). The horizontal dashed line indicates the baseline expression level of normal fed germ-free larvae. Data are presented as mean ± SEM. Statistical significance was determined using a two-tailed unpaired *t* test test. Exact *P* values are indicated on the panel.**

3- Specific role of Lst+ EECs in AA sensing.

This has not formally been demonstrated beyond showing *lst* mRNA expression. Addressing question 1 would allow this. On the other hand, it is not clear to me if the authors have indeed discarded that Lst+ EECs do not respond to dietary sugars.

**Our response to Question 1 provides formal evidence that gut-derived Lst is responsive to amino acid availability in *Drosophila* larvae. To further address whether Lst^+^ EECs also respond to dietary carbohydrates, we utilized a holidic diet to manipulate carbohydrates (sucrose in this case) concentrations. We found that a 50% reduction in sucrose had no significant effect on the expression of gut-derived *Lst*. However, the complete removal of sucrose (0%) from the diet resulted in a mild induction of *Lst* expression (Fig. R3). These results reveal a hierarchical sensitivity to nutrients: while gut-derived Lst is capable of responding to sugar depletion, it is far more sensitive to amino acid fluctuations. This distinction reinforces our model of Lst as a primary mediator of the systemic response to amino acid malnutrition in larvae while it is engaged in metabolic adaptation in response to carbohydrates malnutrition at the adult stage. We have added a section to our Discussion to further address this hierarchical nutrient sensitivity (line 395-400) and its potential physiological implications for larval adaptive growth.**

**Figure R3. Sugar effect on gut-derived *Lst* expression in larvae**

**(A) Relative transcript levels of gut-derived *Lst* analyzed by qRT-PCR in germ-free larvae reared under LAAHD supplemented with 100%, 50%, or 0% sucrose. Data are presented as mean ± SEM. Statistical significance was determined using a one-way ANOVA followed by Tukey’s multiple comparisons. Exact *P* values are indicated on the panel.**

4- The data in Figure 1B and C need clarification: In addition to heterozygotes, Lst whole mutants are also shown in Fig,1B. And they appear to have normal development regardless of the presence or absence of microbiota. However, in Figure 1C, the germ-free whole mutants show high developmental lethality. Similar comparisons are done throughout the manuscript but not clearly explained.

**We apologize for the confusion regarding the phenotypes observed in our study. To clarify, we initially reported that the Lst^1^ null mutant (Lst^−/−^) displays high embryo-to-adult lethality. However, under normal feeding conditions, the surviving Lst^−/−^ animals exhibited developmental timing comparable to that of controls.**

**To determine whether *Lst* is specifically required for viability under malnutrition, we adopted a trans-heterozygous strategy by crossing the Lst^1^ null allele (referred to as Lst^−/−^) with a second allele carrying a CRIMIC insertion within the Lst locus, located at the beginning of the sole intron of the *Lst* gene (Lst*^CR01853^*, hereafter referred to as Lst^CRIMIC^). To bypass potential early embryonic defects, we specifically selected first-instar (L1) larvae of each genotype and monitored larval-to-adult viability. Strikingly, both Lst^−/−^ and Lst*^−/CRIMIC^* larvae displayed survival rates comparable to controls once they had reached the L1 stage (Fig. 1G). These results indicate that the reduced viability initially observed in Lst*^−/−^* animals (Fig. 1C) does not reflect post-embryonic lethality but most likely results from embryonic lethality and/or reduced fertilization/oogenesis efficiency, an issue unrelated to the scope of our study.**

**Importantly, under malnutrition conditions, both mutant genotypes exhibited significantly increased lethality together with accelerated developmental timing (Fig. 1F, H–I). These findings strengthen our conclusion that Lst is a critical regulator of adaptive larval growth, restraining developmental growth in order to preserve larval viability during nutrient stress.**

5- Missing control conditions. There is a general lack of controls in multiple experiments throughout the paper. For example: In Figure 3: Controls including normally fed and non-LP malnourished conditions is necessary. But the same applies to several subsequent figures.

**We apologize for the lack of clarity regarding our control conditions. Our results demonstrate that under normal feeding conditions, gut-derived Lst expression is not induced in either germ free or *Lp*-associated larvae. Furthermore, knocking down *Lst* specifically in EECs—or utilizing *Lst* mutants—does not significantly impact systemic growth when animals are reared on a standard diet or a high amino acid holidic diet (HAAHD). Because the Lst signalling axis is essentially quiescent under nutrient-rich conditions, our study focuses on the under-nutrition state where this pathway is robustly activated. As detailed in our reply to Question 8 below, the phenotypes we describe are strictly dependent on amino acid malnutrition. To provide the necessary physiological context, we have ensured that the relevant 'normally fed' controls are represented in all the supplemental figures of the revised manuscript.**

6- Validation of RNAi lines: It would be important to show Lst knock down (protein) upon EEC overexpression of *lst*-RNAi.

**As suggested, we performed immunostaining to validate the *Lst*-RNAi lines. We observed a marked reduction in Lst protein levels in both RNAi lines (Fig. S3B–C), confirming their efficacy for tissue-specific knockdown. We now also include immunostaining in *Lst^-/-^* mutants confirming the specificity of our anti-Lst antibody (Fig.S3A).**

7- Figure 4: How does the gut protein and Lst+ EEC activity look like in the Low versus high AA diet?

**As our response to Comment 1 states, our new Lst-staining results presented in Fig. 3A-B validate that *Lst* mRNA levels serve as a reliable proxy for Lst protein production and secretion. Because the physiological states of undernourished germ-free larvae and larvae on a low amino acid holidic diet are functionally analogous—both representing a state of nutrient restriction—we utilized the transcriptional profiles from the new Figure 5 (previously Figure 4) onwards to reflect this induction. Our data consistently demonstrate that *Lst* transcripts are robustly induced by nutrient scarcity (Low amino acids) and suppressed under normal-fed conditions (High amino acids).**

8- Figure 4: controls in these experiments are necessary. For example, what is the pupariation time in animals fed a HAAHD and normal diet for the relevant experiments in Figure 4?

**Consistent with our observations under normal feeding conditions, neither *Lst* deficiency nor the knockdown of *Lst* in EECs altered pupariation timing of animals reared on a high amino acid holidic diet (HAAHD). Because these developmental parameters remained unchanged, this data was not included in the original manuscript. As suggested, we have now included the developmental timing for animals reared on HAAHD in Figure (S5B–C, F–G).**

9- Functional interaction between Lst and Ins signalling. This section of the manuscript is very important but very underdeveloped. Conclusions rely largely on correlation rather than genetic interaction experiments. Genetic experiments on gain or loss of function of Insulin signaling, TOR signaling etc should be done in the context of Lst genetic manipulation and assessing the impact to Lst loss of function phenotypes.

**We agree that establishing a functional link between Lst and insulin/insulin-like signalling will strengthen our model. As suggested, we performed genetic epistasis experiments to verify this mechanism. We found that blocking insulin signalling in the EECs—via either *InR*-RNAi or overexpression of a dominant-negative InR (*InR^DN^*)—resulted in a growth phenotype that mirrors the loss of *Lst* per se (Fig. 7M–N). These results indicate that Lst acts downstream of InR in this context. Coupled with our previous functional data, this new result provides a robust mechanistic framework where systemic insulin levels regulate Lst expression in the EECs via InR/FoxO axis.**

10- Regulation of Limostatin in EECs by systemic rather than local nutrient signals. To support this conclusion, the authors should show that AA sensing directly by EE cells is not playing a role here. This could be achieved by inhibition of AA transporters, including slim fast but also other transporters of AA produced by *Lp* would be relevant and assessment of the impact on Lst expression and Lst secretion from EECs in the gut.

**We appreciate the reviewer’s suggestion to examine whether direct amino acid uptake and sensing within EECs contributes to *Lst* regulation. To address this, we first assessed the expression of the cationic amino acid transporter slimfast (*slif*) in the midgut. Our qPCR analysis revealed that *slif* transcript levels are not significantly altered by amino acid deprivation (Fig.1RA – also provided above to address reviewer #2 point 2). Importantly, the targeted knockdown of *slif* specifically in EECs failed to induce *Lst* expression, suggesting that slif-mediated amino acid uptake is not a primary driver of *Lst* regulation in this context (Fig.1RB – also provided above to address reviewer #2 point 2). While we acknowledge that other amino acid transporters (over 20 characterized in *Drosophila*) and sensors (beyond TORC1 and Gcn2) could potentially contribute to cell-autonomous sensing, our data support a model in which gut-derived Lst is primarily controlled by fat body-mediated nutrient sensing and systemic insulin signalling via the InR/FoxO axis. We have expanded our discussion (line 421-423) to address the potential contribution of other EEC-resident transporters which cannot be entirely ruled out while emphasizing the dominance of this systemic feedback loop.**

**Figure R1. Effects of slif-mediated amino acid transport on gut-derived *Lst* expression**

**(A) Relative transcript levels of gut-derived *slif* analyzed by qRT-PCR in germ-free larvae reared under LAAHD (*n* = 6).**

**(B) Relative transcript levels of gut-derived *Lst* analyzed by qRT-PCR in undernourished germ-free larvae with EEC-specific *slif* knockdown (*EEC>UAS-slif* RNAi) compared to their respective GD controls (*n* = 5).**

**In (A, B), data are presented as mean ± SEM. Statistical significance was determined using a two-tailed unpaired *t* test. Exact *P* values are indicated on the respective panels.**

11- Regulation of CNS Ins and downstream signaling by EEC Lst. The evidence for this should be solidified. For example, showing representative images of brain Insulin and also read outs of insulin signaling activity upon Lst knockdown in EECs.

**Following the reviewer’s suggestion, we performed immunostaining for dIlp2 in the larval brain. Upon *Lst* knockdown in EECs, we observed a significant reduction in dIlp2 accumulation within the insulin-producing cells compared to controls (Fig. 8G), which is consistent with an increased dIlp2 secretion and the observed elevated levels of circulating dIlp2 detected in the hemolymph (Fig. 8H). In addition, to further test systemic insulin signalling activity, we measured the transcript levels of *InR*, a well-established (repressed) transcriptional readout of insulin signalling. We found that *InR* expression was significantly downregulated in the fat body, with a trend to a reduction also observed in the carcass, upon *Lst* knockdown in EECs (Fig. 8I–J), further confirming that the knockdown of gut-derived *Lst* leads to elevated systemic insulin activity across peripheral tissues.**

**– – – – – –**

**Reply to Reviewer #4**

Reviewer #4:

As animals develop their success is tied to multiple factors, including their ability to respond to stress. Changes in nutrient availability, especially protein/amino acid restriction, profoundly impact development and the developmental plasticity of animals to alter growth trajectories in response is critical to survival and normal development. In addition to host metabolic, developmental, and immune responses to this process, across animals the microbiome is important in developmental plasticity especially under nutrient limiting conditions. In the model of *Drosophila melanogaster*, protein restriction triggers a reduction in growth, that is worsened in animals without a microbiome. Prior work has shown that the microbiome is able to rescue this growth inhibition when larvae feed on low protein diets and the authors of this manuscript have done extensive work studying the impacts of one strain, *Lactobacillus plantarum^WJL^*, in this response. Previously another lab identified limostatin as a fasting-induced hormone produced in the adult fly brain to suppresses dILP production and release. In the current manuscript, the authors identify a developmental role of limostatin to larvae and show that it is also expressed by a subset of enteroendocrine cells in the anterior midgut in response to amino acid restriction.

Evidence to support manuscript conclusions:

- limostatin (Lst) is up-regulated in larvae raised under malnutrition conditions (extreme low protein (low yeast) of low yeast and microbiome (*L. plantarum^WJL^*) depletion

- Lst+/- and Lst-/- larvae exhibit normal growth in both malnourished and normal conditions

- Using Lst-gal4>GFP observe expression in larval corpora cardiaca, but also in enteroendocrine cells (co-localize with prospero+ cells) in anterior midgut just before the copper cell region

- using tissue specific drivers they show that the effect of Lst is independent of tissue origin

- using a holidic diet they explicitly tested the contribution of specific amino acids, focused on those produced by *L. plantarum^WJL^* (His, Lys, Met, Phe, Thr, Asp)

- using fly mutants and tissue-specific RNAi they show that Lst induction in response to AA limitation occurs independently of canonical intracellular AA sensors in enteroendocrine cells, and is regulated by systemic insulin signaling and that FoxO activity is required for Lst induction

Altogether the authors provide a strong study that uses multiple mechanisms to show a developmental role for limostatin and its role in inter-organ communication to assess nutrient status and support developmental plasticity during times of nutrient stress. This mechanism includes nutrient assimilation by enterocytes, nutrient sensing in the fat body, dILP production in insulin producing cells, and signaling feedback on enteroendocrine cells via insulin signaling. The text is logical and clear and the discussion nicely contextualizes their results, while also defining unanswered questions, such as how limostatin is secreted to the hemolymph and induces dILP2.

One aspect that could maybe use some additional discussion is how specific would this response be to *Lp^WJL^* - is this more broadly a response to microbiome, does limostatin have a role in response to the microbiome outside of response to *Lp^WJL^*. While diet amino acids and *Lp^WJL^* AAs were tested - how does this relate to other microbiome members or even *Lp* strains outside of *Lp^WJL^*.

**We thank the reviewer for this constructive suggestion. We agree that contextualizing the microbial response enhances the broader impact of our findings. Importantly, the suppression of gut-derived *Lst* is not unique to *Lp^WJL^*. We found that another strain of *Lp*, *Lp^NC8^*, and *Acetobacter pomorum^WJL^*, another core member of the *Drosophila* microbiota, similarly downregulates *Lst* expression under malnutrition (Fig.R4 and Fig.S5H). This demonstrates that the Lst response is engaged upon systemic sensing of amino acid of microbial origin rather than to the sensing of a strain/species-specific feature. We have now included the *Ap* data to the revised MS and provide the *Lp^NC8^* data for this reviewer.**

**Figure R4. Microbial regulation of gut-derived *Lst* expression**

**(A) Relative transcript levels of gut-derived *Lst* analyzed by qRT-PCR in germ-free and *Lp^NC8^*-associated larvae reared under LAAHD (*n* = 6). Data are presented as mean ± SEM. Statistical significance was determined using a two-tailed unpaired t test. Exact *P* value is indicated on the panel.**

How likely would a larvae encounter such malnutritive conditions? I think the links to other animals are nicely discussed, but is this relevant outside of this specific association? This needn't be addressed experimentally, but some discussion could make the results more broadly relevant and interesting.

**In their natural environment such as decaying fruit, Drosophila larvae frequently face protein content fluctuations. The microbiome with its ability to produce amino acid serves as a critical nutritional buffer in these contexts, and the Lst-insulin feedback loop allows larvae to tune their growth trajectories to the nutrient yield provided by their environment and their microbial partners. We have expanded the Discussion to include these points, which strengthens the ecological relevance of our physiological model (line 369-373).**

Minor critiques:

L142 and throughout - keep units consistent g/L vs g/L-

If possible reducing some of the abbreviations would be nice and helpful to a broader audience.

**We have standardized the units (g/L) throughout the manuscript to ensure consistency and avoided the use of non-standard abbreviations (CC/GF/FB/AA).**
