# Supplementary material for: A nutrient-sensitive enterokine coordinates developmental plasticity through inter-organ signaling
Source: PLoS Biol. 2026 Aug 11;24(8):e3003911. doi: 10.1371/journal.pbio.3003911 (PMC13460628; doi:10.1371/journal.pbio.3003911)
Supplement: Peer review history

# Editor report: Decision Letter 0

**DOI:** 10.1371/journal.pbio.3003911.r001

**Authors:** Ines Alvarez-Garcia

23 Nov 2025

Dear Dr Leulier,

Thank you for submitting your manuscript entitled "A nutrient-sensitive enterokine coordinates developmental plasticity through inter-organ signalling" for consideration as a Research Article by PLOS Biology.

Your manuscript has now been evaluated by the PLOS Biology editorial staff as well as by an academic editor with relevant expertise and I am writing to let you know that we would like to send your submission out for external peer review.

However, before we can send your manuscript to reviewers, we need you to complete your submission by providing the metadata that is required for full assessment. To this end, please login to Editorial Manager where you will find the paper in the 'Submissions Needing Revisions' folder on your homepage. Please click 'Revise Submission' from the Action Links and complete all additional questions in the submission questionnaire.

Once your full submission is complete, your paper will undergo a series of checks in preparation for peer review. After your manuscript has passed the checks it will be sent out for review. To provide the metadata for your submission, please Login to Editorial Manager (https://www.editorialmanager.com/pbiology) within two working days, i.e. by Nov 25 2025 11:59PM.

If your manuscript has been previously peer-reviewed at another journal, PLOS Biology is willing to work with those reviews in order to avoid re-starting the process. Submission of the previous reviews is entirely optional and our ability to use them effectively will depend on the willingness of the previous journal to confirm the content of the reports and share the reviewer identities. Please note that we reserve the right to invite additional reviewers if we consider that additional/independent reviewers are needed, although we aim to avoid this as far as possible. In our experience, working with previous reviews does save time.

If you would like us to consider previous reviewer reports, please edit your cover letter to let us know and include the name of the journal where the work was previously considered and the manuscript ID it was given. In addition, please upload a response to the reviews as a 'Prior Peer Review' file type, which should include the reports in full and a point-by-point reply detailing how you have or plan to address the reviewers' concerns.

During the process of completing your manuscript submission, you will be invited to opt-in to posting your pre-review manuscript as a bioRxiv preprint. Visit http://journals.plos.org/plosbiology/s/preprints for full details. If you consent to posting your current manuscript as a preprint, please upload a single Preprint PDF.

Feel free to email us at plosbiology@plos.org if you have any queries relating to your submission.

Kind regards,

Ines

—

Ines Alvarez-Garcia, PhD

Senior Editor

PLOS Biology

ialvarez-garcia@plos.org

# Peer review: Decision Letter 1

**DOI:** 10.1371/journal.pbio.3003911.r002

**Authors:** Ines Alvarez-Garcia

12 Jan 2026

Dear Dr Leulier,

Thank you for your patience while your manuscript entitled "A nutrient-sensitive enterokine coordinates developmental plasticity through inter-organ signalling" was peer-reviewed at PLOS Biology. Please also accept again my apologies for the delay in sending you our decision. The manuscript has now been evaluated by the PLOS Biology editors, an Academic Editor with relevant expertise, and by four independent reviewers.

The reviews are attached below. As you will see, the reviewers find the conclusions interesting and worth pursuing, but they also raise several issues that would need to be addressed, some of them experimentally. Reviewer 1 has several suggestions to improve the figures and also thinks the controls for genetic background should be discussed, along with the limitations in the discussion. Reviewer 2 asks for a better description of the developmental stage of the animals and mentions that an alternative explanation for the EECs regulation should be considered, and test whether constitutive activation of TOR signalling in the fat body is sufficient to block lst induction upon AA restriction. Reviewer 3 mentions that Lst positive EEC activity or the haemolymph hormone should be assessed to strengthen the results, and confirm that these EECs don’t respond to dietary sugars, adding missing controls, among other points. Finally, Reviewer 4 asks for additional discussion of the specificity of the response to LpWJL.

In light of the reviews, we would like to invite you to revise the work to thoroughly address the reviewers' reports. Please note that we cannot make a decision about publication until we have seen the revised manuscript and your response to the reviewers' comments. Your revised manuscript is likely to be sent for further evaluation by all or a subset of the reviewers.

In addition to these revisions, you will need to complete some formatting changes, which you will receive in a follow up email. A member of our team will be in touch with a set of requests shortly.

We expect to receive your revised manuscript within 3 months. Please email us (plosbiology@plos.org) if you have any questions or concerns, or would like to request an extension.

At this stage, your manuscript remains formally under active consideration at our journal; please notify us by email if you do not intend to submit a revision so that we may withdraw it.

**IMPORTANT - SUBMITTING YOUR REVISION**

Your revisions should address the specific points made by each reviewer. Please submit the following files along with your revised manuscript:

1. A 'Response to Reviewers' file - this should detail your responses to the editorial requests, present a point-by-point response to all of the reviewers' comments, and indicate the changes made to the manuscript.

*NOTE: In your point-by-point response to the reviewers, please provide the full context of each review. Do not selectively quote paragraphs or sentences to reply to. The entire set of reviewer comments should be present in full and each specific point should be responded to individually, point by point.

You should also cite any additional relevant literature that has been published since the original submission and mention any additional citations in your response.

2. In addition to a clean copy of the manuscript, please also upload a 'track-changes' version of your manuscript that specifies the edits made. This should be uploaded as a "Revised Article with Changes Highlighted" file type.

3. Resubmission Checklist

When you are ready to resubmit your revised manuscript, please refer to this resubmission checklist: https://plos.io/Biology_Checklist

To submit a revised version of your manuscript, please go to https://www.editorialmanager.com/pbiology/ and log in as an Author. Click the link labelled 'Submissions Needing Revision' where you will find your submission record.

Please make sure to read the following important policies and guidelines while preparing your revision and fulfil the editorial requests:

a) *PLOS Data Policy*

Please note that as a condition of publication PLOS' data policy (http://journals.plos.org/plosbiology/s/data-availability) requires that you make available all data used to draw the conclusions arrived at in your manuscript. If you have not already done so, you must include any data used in your manuscript either in appropriate repositories, within the body of the manuscript, or as supporting information (N.B. this includes any numerical values that were used to generate graphs, histograms etc.). Please also indicate in each figure legend where the data can be found. For an example see here: http://www.plosbiology.org/article/info%3Adoi%2F10.1371%2Fjournal.pbio.1001908#s5

b) *Published Peer Review*

Please note while forming your response, if your article is accepted, you may have the opportunity to make the peer review history publicly available. The record will include editor decision letters (with reviews) and your responses to reviewer comments. If eligible, we will contact you to opt in or out. Please see here for more details:

https://blogs.plos.org/plos/2019/05/plos-journals-now-open-for-published-peer-review/

c) *Blot and Gel Data Policy*

Please provide the original, uncropped and minimally adjusted images supporting all blot and gel results reported in an article's figures or Supporting Information files. We will require these files before a manuscript can be accepted so please prepare them now, if you have not already uploaded them. Please carefully read our guidelines for how to prepare and upload this data: https://journals.plos.org/plosbiology/s/figures#loc-blot-and-gel-reporting-requirements

d) *Blurb*

Please also provide a blurb which (if accepted) will be included in our weekly and monthly Electronic Table of Contents, sent out to readers of PLOS Biology, and may be used to promote your article in social media. The blurb should be about 30-40 words long and is subject to editorial changes. It should, without exaggeration, entice people to read your manuscript. It should not be redundant with the title and should not contain acronyms or abbreviations. For examples, view our author guidelines: https://journals.plos.org/plosbiology/s/revising-your-manuscript#loc-blurb

e) *Protocols deposition*

To enhance the reproducibility of your results, we recommend that if applicable you deposit your laboratory protocols in protocols.io, where a protocol can be assigned its own identifier (DOI) such that it can be cited independently in the future. For instructions see: https://journals.plos.org/plosbiology/s/submission-guidelines#loc-materials-and-methods

Thank you again for your submission to our journal. We hope that our editorial process has been constructive thus far, and we welcome your feedback at any time. Please don't hesitate to contact us if you have any questions or comments.

Sincerely,

Ines

--

Ines Alvarez-Garcia, PhD

Senior Editor

PLOS Biology

ialvarez-garcia@plos.org

------------------------------------

Reviewers' comments

Rev. 1:

Bai et al discover a role of anterior midgut-localized Limostatin-producing cells in the regulation of Drosophila larval development by using a malnutrition paradigm that more closely resembles low nutrient conditions in the wild. They find that Limostatin represses developmental progression, conditional on the nutrient composition of the growth medium, with low nutrients causing upregulation of Lst and slowing of development. The authors find that the bacterium LpWJL is sufficient to inhibit Lst production, consistent with its role in providing additional nutrients to the malnutrition medium. While Limostatin is known as a decretin in adults, the role in larval development has only been presumed, and the localization of Lst-producing cells has been thought to be in the corpora cardiaca. The authors use Gal4 drivers and antibodies to localize and characterize the functional importance of the new cell population that they discovered in the midgut. The paper is very clearly written and the data well-presented. I think the evidence is strong enough for publication.

I have some minor comments that I think the authors should address through writing changes in the manuscript.

1. Fig. 1. Since the unbiased RNAseq approach was used to identify Lst as an interesting candidate, a full presentation of the data would provide readers with a more unbiased perspective on the data. A volcano plot or other depiction of the complete dataset would suffice. DESeq2 could be used for the statistical test. If the data are messy or there are many other genes that are differentially upregulated in GF vs Lp colonized flies, this additional analysis could be put in the supplement and the rationale for pursuing Lst could be elaborated.

2. Fig. 2. A+B) please show zoomed out images of the entire dissected CC and gut for spatial reference.

3. Please discuss the controls for genetic background in the main text of the paper. For example, when a Gal4 line and a UAS line are crossed, were controls used for each background? In Fig. 3 for instance, there is a Control 1 and a Control 2 in some panels. Please explain these further and provide the rationale in the main text or methods. I think it is particularly important for trainees who read Drosophila papers to appreciate how genetic background is accounted for experimentally -- either with backcrossing, with measurements of the parental phenotypes, or with inducible mutations. I think it would also be helpful to discuss the limitations in the discussion.

4. I'm not fully convinced that the small subset of cells in the anterior midgut that show high Lst expression are a signaling center composed of the only causal cells for the fly developmental phenotype. The authors acknowledge that the Gal4 drivers could have off-target effects, and I appreciate that. Could these cells just be mirroring the effect from the causal cells, which could be located in another region that the Gal4 driver targets? Could there be many dispersed cells expressing Lst at lower levels to produce the organismal growth effect? I am not asking for additional experiments but think that the Gal4 caveats and their implications should be discussed a bit more.

5. I am curious from an evolutionary biology perspective why the effective Lst is expressed specifically in the EECs in the anterior midgut rather than in another signaling center such as the CC? Is there a potential integration of the local nutrient status in the gut with systemic nutrient status? Why should it work this way versus in an alternative way? Is there a particular developmental reason based on the cell lineages? Some discussion of these questions would strengthen the manuscript.

. I think it would be worth including the model figure in the main text if there is room.

line 207: in gut --> in the gut

line 208: majors --> major

Rev. 2: Fumiaki Obata – note that this reviewer has signed the review.

In this study, the authors address the endocrine mechanisms of adaptation to malnutrition in Drosophila larvae. They identified a previously unrecognized role of the peptide hormone Limostatin (Lst) in regulating larval developmental plasticity in response to amino acid (AA) availability. While Lst has been characterized primarily as a hormone produced by the corpora cardiaca in adult flies, this work convincingly demonstrates that, in larvae, Lst is secreted from a small population of midgut enteroendocrine cells. Through the use of chemically defined diets and well-controlled tissue-specific genetic manipulations, the authors delineate an inter-organ endocrine circuit linking AA sensing to systemic insulin signaling and developmental progression. The experiments are carefully executed, and the data consistently support the proposed model. Overall, this is a clearly presented study that provides new insight into how nutritional status is integrated at the organismal level to regulate developmental pace.

I have only minor comments, which the authors may find helpful for improving the manuscript. Addressing these points would further strengthen the study, but additional experiments are not strictly required.

1. The developmental stage of animals are not well described. It might be better to clearly specify the developmental stage of larvae, especially given that dietary conditions or Lst manipulation are expected to alter developmental timing. Were samples collected at matched chronological time points or at matched developmental stages?

2. While the proposed model emphasizes an indirect relay via the fat body for the regulation of EECs, it is also possible that EECs directly sense amino acids (independently of GCN2/TOR). For example, does slif RNAi in EECs induce lst expression? In the model, there might be a direct arrow from "Low AA" to EEC. In addition, it would be informative to test whether constitutive activation of TOR signaling in the fat body (e.g. by Rheb overexpression) is sufficient to block lst induction upon AA restriction.

3. In the model, FoxO is directly regulating lst expression. Is there evidence supporting this, such as the presence of FoxO-binding motifs in the regulatory region upstream of the lst gene?

4. Given that only ~10 EECs express lst, further characterization of this population would be of interest. For example, analysis of available single-cell RNA-seq datasets could reveal whether these cells express additional distinctive markers or signaling components.

5. Please check the notation of Limostatin throughout the manuscript, as there are inconsistencies in several figures.

(Fumiaki Obata)

Rev. 3:

This manuscript by Bai et al describes the presence of an midgut enteroendocrine source of the hormone Limostatin (Lst), which regulates organismal developmental control in response to dietary nutrients (AA in particular) and the microbiota in Drosophila larva.

The expression of Lst in the larval midgut is regulated by nutrients through Insulin signaling and it, in turn, controls Insulin signal production by the brain. The authors propose that low nutrient condition in the absence of microbiota leads to low insulin signaling in enteroendocrine cells (EECs) which, through FOXO, induces expression of Lst in EECs. Lst in turn further inhibits brain Ins.

This is, to my knowledge, the first report on a functional role of the larval midgut and EECs in the regulation of whole-body physiology in Drosophila. As such, this work presents exciting findings of great conceptual novelty and biological significance.

However, there is critical work that needs to be done, including important controls, to fully support the main claims of the study and to also strengthen its significant and impact in the field. I believe the suggested work below is also important to bring the study into publishable form.

1- Regulation of by dietary aminoacids, microbiota and Ins signaling.

In most, if not all cases, throughout the manuscript, this is concluded from assessment of mRNA expression of Lst in whole midguts. This work needs to be complemented by the used of antibody and immunostaining to visualise the meaning of the mRNA levels observed. Also, levels of protein in the gut need to be followed by either assessment of Lst positive EEC activity (for example thorugh CaLexA of GCamp signaling) or assessment of haemolymph hormone, which is very difficult, so I suggest the former. Only then, the authors will be able to conclude what is happening with Lst in the various conditions studied.

2- EEC Lemostatin has a developmental only role.

As part of their conclusions, the authors propose that Lst from EEC works in development, but the CC hormone works in adults. Have they looked at the expression or role of Lst in adult EECs at all?

3- Specific role of Lst+ EECs in AA sensing.

This has not formally been demonstrated beyond showing lst mRNA expression. Addressing question 1 would allow this. On the other hand, it is not clear to me if the authors have indeed discarded that Lst+ EECs do not respond to dietary sugars.

4- The data in Figure 1B and C need clarification: In addition to heterozygotes, Lst whole mutants are also shown in Fig,1B. And they appear to have normal development regardless of the presence or absence of microbiota. However, in Figure 1C, the germ-free whole mutants show high developmental lethality. Similar comparisons are done throughout the manuscript but not clearly explained.

5- Missing control conditions. There is a general lack of controls in multiple experiments throughout the paper. For example: In Figure 3: Controls including normally fed and non-LP malnourished conditions is necessary. But the same applies to several subsequent figures.

6- Validation of RNAi lines: It would be important to show Lst knock down (protein) upon EEC overexpression of lst-RNAi.

7- Figure 4: How does the gut protein and Lst+ve EEC activity look like in the Low versus high AA diet?

8- Figure 4: controls in these experiments are necessary. For example, what is the pupariation time in animals fed a HAAD and normal diet for the relevant experiments in Figure 4?

9- Functional interaction between Lst and Ins signalling. This section of the manuscript is very important but very underdeveloped. Conclusions rely largely on correlation rather than genetic interaction experiments. Genetic experiments on gain or loss of function of Insulin signaling, TOR signaling etc should be done in the context of Lst genetic manipulation and assessing the impact to Lst loss of function phenotypes.

10- Regulation of Lemostatin in EECs by systemic rather than local nutrient signals. To support this conclusion, the authors should show that AA sensing directly by EE cells is not playing a role here. This could achieved by Inhibition of AA transporters, including slim fast but also other transporters of AA produced by Lp would be relevant and assessment of the impact on Lst expression and Lst secretion from EECs in the gut.

11- Regulation of CNS Ins and downstream signaling by EEC Lst. The evidence for this should be solidified. For example, showing representative images of brain Insulin and also read outs of insulin signaling activity upon Lst knockdown in EECs.

Rev. 4:

As animals develop their success is tied to multiple factors, including their ability to respond to stress. Changes in nutrient availability, especially protein/amino acid restriction, profoundly impact development and the developmental plasticity of animals to alter growth trajectories in response is critical to survival and normal development. In addition to host metabolic, developmental, and immune responses to this process, across animals the microbiome is important in developmental plasticity especially under nutrient limiting conditions. In the model of Drosophila melanogaster, protein restriction triggers a reduction in growth, that is worsened in animals without a microbiome. Prior work has shown that the microbiome is able to rescue this growth inhibition when larvae feed on low protein diets and the authors of this manuscript have done extensive work studying the impacts of one strain, Lactobacillus plantarumWJL, in this response. Previously another lab identified limostatin as a fasting-induced hormone produced in the adult fly brain to suppresses dILP production and release. In the current manuscript, the authors identify a developmental role of limostatin to larvae and show that it is also expressed by a subset of enteroendocrine cells in the anterior midgut in response to amino acid restriction.

Evidence to support manuscript conclusions:

- limostatin (Lst) is up-regulated in larvae raised under malnutrition conditions (extreme low protein (low yeast) of low yeast and microbiome (L. plantarumWJL) depletion

- Lst+/- and Lst-/- larvae exhibit normal growth in both malnourished and normal conditions

- Using Lst-gal4>GFP observe expression in larval corpora cardiaca, but also in enteroendocrine cells (co-localize with prospero+ cells) in anterior midgut just before the copper cell region

- using tissue specific drivers they show that the effect of Lst is independent of tissue origin

- using a holidic diet they explicitly tested the contribution of specific amino acids, focused on those produced by L. plantarum WJL (His, Lys, Met, Phe, Thr, Asp)

- using fly mutants and tissue-specific RNAi they show that Lst induction in response to AA limitation occurs independently of canonical intracellular AA sensors in enteroendocrine cells, and is regulated by systemic insulin signaling and that FoxO activity is required for Lst induction

Altogether the authors provide a strong study that uses multiple mechanisms to show a developmental role for limostatin and its role in inter-organ communication to assess nutrient status and support developmental plasticity during times of nutrient stress. This mechanism includes nutrient assimilation by enterocytes, nutrient sensing in the fat body, dILP production in insulin producing cells, and signaling feedback on enteroendocrine cells via insulin signaling. The text is logical and clear and the discussion nicely contextualizes their results, while also defining unanswered questions, such as how limostatin is secreted to the hemolymph and induces dILP2.

One aspect that could maybe use some additional discussion is how specific would this response be to LpWJL - is this more broadly a response to microbiome, does limosatin have a role in response to the microbiome outside of response to LpWJL. While diet amino acids and LpWJL AAs were tested - how does this relate to other microbiome members or even Lp strains outside of LpWJL. How likely would a larvae encounter such malnutritive conditions? I think the links to other animals are nicely discussed, but is this relevant outside of this specific association? This needn't be addressed experimentally, but some discussion could make the results more broadly relevant and interesting.

Minor critiques:

L142 and throughout - keep units consistent g/L vs g/L-

If possible reducing some of the abbreviations would be nice and helpful to a broader audience.

# Author comment: Author response to Decision Letter 2

**DOI:** 10.1371/journal.pbio.3003911.r003

2 Jun 2026

Attachment Submitted filename: Reply to the reviewers .docx

# Peer review: Decision Letter 2

**DOI:** 10.1371/journal.pbio.3003911.r004

**Authors:** Ines Alvarez-Garcia

19 Jun 2026

Dear Dr Leulier,

Thank you for your patience while we considered your revised manuscript entitled "A nutrient-sensitive enterokine coordinates developmental plasticity through inter-organ signalling" for publication as a Research Article at PLOS Biology. This revised version of your manuscript has been evaluated by the PLOS Biology editors, the Academic Editor and two of the original reviewers.

Based on the reviews (attached below), we are likely to accept this manuscript for publication, provided you satisfactorily address the data and other policy-related requests stated below my signature.

As you address these items, please take this last chance to review your reference list to ensure that it is complete and correct. If you have cited papers that have been retracted, please include the rationale for doing so in the manuscript text, or remove these references and replace them with relevant current references. Any changes to the reference list should be mentioned in the cover letter that accompanies your revised manuscript.

In addition to these revisions, you may need to complete some formatting changes, which you will receive in a follow up email. A member of our team will be in touch with a set of requests shortly. If you do not receive a separate email within a few days, please assume that checks have been completed, and no additional changes are required.

We expect to receive your revised manuscript within two weeks.

To submit your revision, please go to https://www.editorialmanager.com/pbiology/ and log in as an Author. Click the link labelled 'Submissions Needing Revision' to find your submission record. Your revised submission must include the following:

- a cover letter that should detail your responses to any editorial requests, if applicable, and whether changes have been made to the reference list

- a Response to Reviewers file that provides a detailed response to the reviewers' comments (if applicable, if not applicable please do not delete your existing 'Response to Reviewers' file.)

- a track-changes file indicating any changes that you have made to the manuscript.

NOTE: If Supporting Information files are included with your article, note that these are not copyedited and will be published as they are submitted. Please ensure that these files are legible and of high quality (at least 300 dpi) in an easily accessible file format. For this reason, please be aware that any references listed in an SI file will not be indexed. For more information, see our Supporting Information guidelines:

https://journals.plos.org/plosbiology/s/supporting-information

*Published Peer Review History*

Please note that you may have the opportunity to make the peer review history publicly available. The record will include editor decision letters (with reviews) and your responses to reviewer comments. If eligible, we will contact you to opt in or out. Please see here for more details:

https://plos.org/published-peer-review-history/

*Press*

Should you, your institution's press office or the journal office choose to press release your paper, please ensure you have opted out of Early Article Posting on the submission form. We ask that you notify us as soon as possible if you or your institution is planning to press release the article.

*Protocols deposition*

To enhance the reproducibility of your results, we recommend that if applicable you deposit your laboratory protocols in protocols.io, where a protocol can be assigned its own identifier (DOI) such that it can be cited independently in the future. Additionally, PLOS ONE offers an option for publishing peer-reviewed Lab Protocol articles, which describe protocols hosted on protocols.io. Read more information on sharing protocols at https://plos.org/protocols?utm_medium=editorial-email&utm_source=authorletters&utm_campaign=protocols

Please do not hesitate to contact me should you have any questions.

Sincerely,

Ines

--

Ines Alvarez-Garcia, PhD

Senior Editor

ialvarez-garcia@plos.org

PLOS Biology

------------------------------------------------------------------------

DATA POLICY:

You may be aware of the PLOS Data Policy, which requires that all data be made available without restriction: http://journals.plos.org/plosbiology/s/data-availability. For more information, please also see this editorial: http://dx.doi.org/10.1371/journal.pbio.1001797

Note that we do not require all raw data. Rather, we ask that all individual quantitative observations that underlie the data summarized in the figures and results of your paper be made available in one of the following forms:

1) Supplementary files (e.g., excel). Please ensure that all data files are uploaded as 'Supporting Information' and are invariably referred to (in the manuscript, figure legends, and the Description field when uploading your files) using the following format verbatim: S1 Data, S2 Data, etc. Multiple panels of a single or even several figures can be included as multiple sheets in one excel file that is saved using exactly the following convention: S1_Data.xlsx (using an underscore).

2) Deposition in a publicly available repository. Please also provide the accession code or a reviewer link so that we may view your data before publication.

Regardless of the method selected, please ensure that you provide the individual numerical values that underlie the summary data displayed in the following figure panels as they are essential for readers to assess your analysis and to reproduce it:

Fig. 1A-I; Fig. 2D-G; Fig. 4A-H; Fig. 5A-F; Fig. 6A-D; Fig. 7A, C-N; Fig. 8A-F, H-J; Fig. S1A-F; Fig. S4A-G; Fig. S5A-H; Fig. S6A-G and Fig. S8A-H

NOTE: the numerical data provided should include all replicates AND the way in which the plotted mean and errors were derived (it should not present only the mean/average values).

Please also ensure that figure legends in your manuscript include information on WHERE THE UNDERLYING DATA CAN BE FOUND, and ensure your supplemental data file/s has a legend.

Please ensure that your Data Statement in the submission system accurately describes where your data can be found.

------------------------------------------------------------------------

CODE POLICY

Per journal policy, if you have generated any custom code during the course of this investigation, please make it available without restrictions. Please ensure that the code is sufficiently well documented and reusable, and that your Data Statement in the Editorial Manager submission system accurately describes where your code can be found. More information on our Code Policy, what and how to share can be found here: https://journals.plos.org/plosbiology/s/code-availability

Please note that we cannot accept sole deposition of code in GitHub, as this could be changed after publication. However, you can archive this version of your publicly available GitHub code to Zenodo. Once you do this, it will generate a DOI number, which you will need to provide in the Data Accessibility Statement (you are welcome to also provide the GitHub access information). See the process for doing this here: https://docs.github.com/en/repositories/archiving-a-github-repository/referencing-and-citing-content

------------------------------------------------------------------------

Reviewers' discussion

Rev. 2: Fumiaki Obata

The authors have addressed my comments with new experiments and have further strengthened the manuscript. I thank the authors' great efforts. I believe the revised version is suitable for publication.

Rev. 3:

I thank the authors for taking the time to carefully and thoughtfully review their manuscript in line with my suggestions. The addition of protein staining for assessment of Lst production/secretion by EE cells in response to dietary challenges, the distinction between dietary protein vs sugar sensitivity and the developmental versus adult function of Lst positive cells, as well as the further insights into the role of EE cell derived Lst on systemic insulin signalling make this study even stronger and more impactful to the field. Congratulations.

# Author comment: Author response to Decision Letter 3

**DOI:** 10.1371/journal.pbio.3003911.r005

29 Jun 2026

Attachment Submitted filename: Reply_to_the_reviewers__auresp_3.docx

# Editor report: Decision Letter 3

**DOI:** 10.1371/journal.pbio.3003911.r006

**Authors:** Ines Alvarez-Garcia

3 Jul 2026

Dear Dr Leulier,

Thank you for the submission of your revised Research Article entitled "A nutrient-sensitive enterokine coordinates developmental plasticity through inter-organ signalling" for publication in PLOS Biology. On behalf of my colleagues and the Academic Editor, Alex Gould, I am delighted to let you know that we can in principle accept your manuscript for publication, provided you address any remaining formatting and reporting issues. These will be detailed in an email you should receive within 2-3 business days from our colleagues in the journal operations team; no action is required from you until then. Please note that we will not be able to formally accept your manuscript and schedule it for publication until you have completed any requested changes.

Please take a minute to log into Editorial Manager at http://www.editorialmanager.com/pbiology/, click the "Update My Information" link at the top of the page, and update your user information to ensure an efficient production process.

PRESS

We frequently collaborate with press offices. If your institution or institutions have a press office, please notify them about your upcoming paper at this point, to enable them to help maximise its impact. If the press office is planning to promote your findings, we would be grateful if they could coordinate with biologypress@plos.org. If you have previously opted in to the early version process, we ask that you notify us immediately of any press plans so that we may opt out on your behalf.

We also ask that you take this opportunity to read our Embargo Policy regarding the discussion, promotion and media coverage of work that is yet to be published by PLOS. As your manuscript is not yet published, it is bound by the conditions of our Embargo Policy. Please be aware that this policy is in place both to ensure that any press coverage of your article is fully substantiated and to provide a direct link between such coverage and the published work. For full details of our Embargo Policy, please visit http://www.plos.org/about/media-inquiries/embargo-policy/.

Many congratulations and thanks again for choosing PLOS Biology for publication and supporting Open Access publishing. We look forward to publishing your study.

Sincerely,

Ines

--

Ines Alvarez-Garcia, PhD

Senior Editor

PLOS Biology

ialvarez-garcia@plos.org
